# Relationship Between Neutrophil-Lymphocyte Ratio and Treatment Retention in Individuals with Opioid Use Disorder

**DOI:** 10.1192/j.eurpsy.2024.617

**Published:** 2024-08-27

**Authors:** Y. Taylan, M. B. Sönmez

**Affiliations:** ^1^İstanbul Sultanbeyli State Hospital, İstanbul; ^2^Trakya University School of Medicine, Edirne, Türkiye

## Abstract

**Introduction:**

Inflammatory processes may play a role in the pathophysiology of substance use disorders. Chronic opiate use may lead to inflammation, and elevated inflammation markers have been observed in individuals with opioid use disorder (OUD). The Neutrophil-Lymphocyte Ratio (NLR) serves as an indicator of systemic inflammation. NLR can be employed in both diagnosis and treatment monitoring as an inflammatory marker to gauge the severity of OUD.

**Objectives:**

Our aim was to assess the utility of NLR as a marker of chronic inflammation in diagnosing and monitoring treatment in individuals with OUD.

**Methods:**

A total of 200 patients with OUD and 78 healthy control subjects were enrolled in the study. Patients were initially admitted to a 28-day abstinence-based inpatient program and subsequently transitioned to outpatient buprenorphine/naloxone (B/N) maintenance treatment after hospitalization at the Alcohol and Substance Addiction Treatment Center in Trakya University School of Medicine (Edirne, Türkiye). NLR was employed as a measure of systemic inflammation. Blood samples were collected the morning following admission for detoxification. Patients were categorized into two groups: the treatment retention group and the dropout/relapse group based on their 3-month and 12-month follow-up results. Clinical data were obtained from patient records.

**Results:**

At the 3-month follow-up, the median NLR with interquartile range was 1.34 (1.05-1.99) in the treatment retention group (n=112) and 1.72 (1.11-2.46) in the dropout/relapse group (n=88). At the 12-month follow-up, the median NLR with interquartile range was 1.28 (0.88-1.85) in the treatment retention group (n=52) and 1.56 (1.07-2.33) in the dropout/relapse group (n=148). The median NLR in the control group (n=78) was 1.36 (1.12-1.74). According to the 3-month and 12-month follow-up data, the difference between the groups concerning NLR was statistically significant (χ^2^=9.072, p=0.011; χ^2^=11.165, p=0.004; respectively). Pairwise comparisons indicated that patients in the dropout/relapse group had significantly higher baseline NLR values than those in the treatment retention group and healthy controls according to the 3-month (p=0.038 and p=0.019, respectively) and 12-month follow-up data (p=0.012 and p=0.040, respectively). NLR did not differ significantly between the treatment retention and control groups in both follow-ups (p>0.05).

**Conclusions:**

Our findings suggest that elevated baseline NLR is associated with dropout/relapse in OUD, indicating its potential as a marker for treatment follow-up in these patients.

**Disclosure of Interest:**

None Declared

